# Laryngeal Elevation Velocity as a Candidate Dynamic Marker of Swallowing Safety in Postradiotherapy Dysphagia: A Preliminary Exploratory Study

**DOI:** 10.3390/jcm15197523

**Published:** 2026-09-27

**Authors:** Kai-Hsiang Hu, Shu-Wei Tsai, Tammy Tsai, Miyuki Hsing-Chun Hsieh, Yi-Jen Chen, Jenn-Ren Hsiao, Cheng-Chih Huang, Chun-Yen Ou, Chan-Chi Chang, Wei-Ting Lee, Sen-Tien Tsai, Hui-Chen Su, David Shang-Yu Hung

**Affiliations:** 1 Department of Education Center, National Cheng Kung University Hospital, College of Medicine, National Cheng Kung University, Tainan 704, Taiwan; michael880129@gmail.com; 2 Department of Otolaryngology, National Cheng Kung University Hospital, College of Medicine, National Cheng Kung University, Tainan 704, Taiwan; tsaisuwei@gmail.com (S.-W.T.); ivanyjchen@email.ncku.edu.tw (Y.-J.C.); hsiaojr@mail.ncku.edu.tw (J.-R.H.); cs841063@yahoo.com.tw (C.-C.H.); ojy1@mail.ncku.edu.tw (C.-Y.O.); guitarist217@yahoo.com.tw (C.-C.C.); wendelllee92@yahoo.com.tw (W.-T.L.); t602511@mail.ncku.edu.tw (S.-T.T.); 3 Department of Otolaryngology, Tainan Municipal Hospital (Managed by Show Chwan Medical Care Corporation), Tainan 701, Taiwan; 2le168@tmh.org.tw; 4 Institute of Clinical Pharmacy and Pharmaceutical Sciences, College of Medicine, National Cheng Kung University, Tainan 704, Taiwan; s1234567y@gmail.com; 5 Chewing and Swallowing Center, National Cheng Kung University Hospital, College of Medicine, National Cheng Kung University, Tainan 704, Taiwan; i54071212@gs.ncku.edu.tw

**Keywords:** dysphagia, botulinum toxin injection, laryngeal elevation velocity, nasopharyngeal carcinoma, UES opening

## Abstract

**Background/Objectives**: To investigate laryngeal elevation velocity (LEV) as a candidate physiological marker of swallowing safety in post-radiotherapy dysphagia patients with cricopharyngeal muscle dysfunction (CPMD), we examined its association with airway safety and whether the LEV–upper esophageal sphincter (UES) opening relationship differed before versus after botulinum toxin injection, used here as a within-subject physiological perturbation. **Methods**: This retrospective cohort study included 18 post-radiotherapy patients with nasopharyngeal carcinoma (NPC) and CPMD who underwent botulinum toxin injection into the cricopharyngeal muscle (median total dose, 30 U; range, 20–40 U) and completed videofluoroscopic swallowing studies (VFSSs) before and after intervention. Primary outcomes were the Penetration–Aspiration Scale (PAS) and UES opening diameter; secondary outcomes included the Functional Oral Intake Scale (FOIS), laryngeal elevation (LE) and LEV. Associations were evaluated using generalized estimating equations (GEEs) with LEV × treatment interaction terms to assess whether they differed across pre- and post-injection states. **Results**: Following botulinum toxin injection, FOIS scores improved significantly (median, 4 to 5; *p* < 0.001), with concurrent increases in LEV (median, 149.86 to 201.12 C2–4%/s; *p* = 0.027) and UES diameter (median, 21.10 to 23.30 C2–4%; *p* = 0.039); PAS scores and laryngeal elevation displacement did not change significantly. Higher LEV was associated with lower odds of unsafe airway invasion (PAS 5–8) in univariate analysis (odds ratio per 100-unit increase, 0.38; 95% confidence interval [CI], 0.17–0.86; *p* = 0.021). A significant LEV × treatment interaction was observed for UES diameter (*p* = 0.021). **Conclusions**: In post-radiotherapy NPC patients with CPMD, higher LEV was associated with airway safety, and the LEV–UES relationship differed across pre- and post-injection states. Although the study design does not permit conclusions about treatment efficacy, LEV may represent a candidate load-sensitive marker of swallowing safety, warranting further investigation.

## 1. Introduction

Dysphagia is among the most common and disabling long-term complications of radiotherapy for head and neck cancer, and cricopharyngeal muscle dysfunction (CPMD) is a major contributor to this impairment [1,2]. Radiation-induced injury to the pharyngeal constrictors and cricopharyngeal muscle disrupts the finely coordinated neuromuscular sequence of swallowing, producing impaired bolus transit, post-swallow residue, and airway invasion [3]. These physiological deficits carry substantial clinical consequences, including malnutrition, an increased risk of aspiration pneumonia, prolonged feeding-tube dependence, and a marked deterioration in health-related quality of life [4,5]. Nasopharyngeal carcinoma (NPC), which is endemic to East and Southeast Asia and treated principally with radiotherapy or concurrent chemoradiotherapy, is particularly relevant in this regard, as a considerable proportion of survivors develop chronic, fibrosis-related swallowing impairment. Characterizing the biomechanical determinants of swallowing safety in this population is therefore of clear clinical importance.

Swallowing safety depends not only on the extent of laryngeal elevation (LE) but also on the timing and velocity of hyolaryngeal movement [6]. Although LE is traditionally conceptualized as a displacement-based mechanism facilitating airway protection and upper esophageal sphincter (UES) opening, emerging evidence suggests that the velocity of LE may be a more sensitive determinant of effective airway closure [7,8]. Insufficient elevation velocity can prolong pharyngeal bolus dwell time and delay laryngeal vestibule closure (LVC), thereby increasing the risk of airway invasion even when maximal elevation is preserved [9]. Despite growing recognition of laryngeal elevation velocity (LEV) as a dynamic marker of swallowing safety, its physiological and clinical significance remains incompletely defined, particularly in patient populations with altered biomechanical loading conditions [10].

Zhang et al. reported that among patients with acute ischemic stroke, reduced LEV was an independent predictor of aspiration, underscoring the importance of laryngeal kinematics in airway protection. They further demonstrated that for every 10 mm/s decrease in peak LEV, the risk of severe penetration–aspiration increased by 43%, highlighting that LEV, rather than displacement magnitude alone, more accurately reflects swallowing function [6]. Consistent with this concept, another study showed that in patients with severe dysphagia, peak hyoid and laryngeal movement velocities were poorly synchronized with LVC, thereby compromising airway defense [11].

While the association between LEV and swallowing safety is well supported, its relationship with UES opening remains unclear. Beyond airway protection, LEV exerts traction on the relaxed UES to facilitate its expansion [12]. Theoretically, faster LEV may result in more efficient UES opening, thereby improving swallowing efficiency [13]. However, one prior study found that laryngeal elevation (LE), a parameter closely related to LEV, is not significantly correlated with UES expansion, suggesting that LEV may be more specifically linked to airway protection [12]. UES opening is governed by multiple factors, including cricopharyngeal relaxation, laryngeal traction, intrabolus pressure, and pharyngeal contraction; however, the independent role of LEV in populations with altered pharyngeal biomechanics remains poorly characterized [14,15]. Overall, the dynamic interplay between LEV and UES opening lacks a consistent conclusion.

Patients with NPC who undergo radiotherapy (RT) or concurrent chemoradiotherapy are at substantial risk of radiation-induced swallowing impairment. Radiation-induced structural changes, including muscle fibrosis, reduced elasticity, and neural injury, impair the coordinated neuromuscular activity required for laryngeal elevation and UES relaxation, leading to cricopharyngeal muscle dysfunction (CPMD) and severe dysphagia [16]. Among available treatments, endoscopy-guided botulinum toxin injection has gained clinical acceptance due to its minimally invasive nature and efficacy in reducing cricopharyngeal muscle tension [17]. Although our previous study demonstrated that botulinum toxin injection improves Functional Oral Intake Scale (FOIS) scores and UES opening diameter in this population, the kinematic mechanisms underlying these changes and reliable dynamic markers of treatment response remain to be established [17].

Against this background, the primary aim of the present study was to investigate LEV as a candidate physiological biomarker of swallowing safety in NPC patients after RT complicated by CPMD, using endoscopy-guided botulinum toxin injection as a within-subject physiological perturbation that transiently alters cricopharyngeal loading conditions. Within this framework, the specific objectives were (1) to examine whether LEV is associated with the Penetration–Aspiration Scale (PAS), thereby clarifying its potential contribution to swallowing safety; and (2) to compare pre- and post-perturbation changes in LE, LEV, and UES opening to explore their dynamic relationship. Botulinum toxin was therefore treated as a perturbation model rather than as the primary exposure or as a treatment whose efficacy this study sought to establish.

## 2. Materials and Methods

### 2.1. Participants

This study was designed as a retrospective cohort study conducted at a tertiary medical center. Eligible participants were adults aged 18 years or older with a confirmed diagnosis of NPC who had completed radiotherapy or concurrent chemoradiotherapy at least one year prior to enrollment. The minimum interval of one year was required to ensure that acute radiation-induced effects had resolved and that observed dysphagia represented chronic, fibrosis-mediated impairment. All participants were diagnosed with CPMD by an otolaryngologist, defined as an anteroposterior UES opening diameter of less than 10 mm on videofluoroscopic swallowing study (VFSS), consistent with established radiographic criteria [17,18]. Additionally, all participants had undergone conservative swallowing rehabilitation without satisfactory improvement.

Following botulinum toxin injection, all participants continued their pre-existing speech-language pathologist-directed swallowing rehabilitation until the post-treatment VFSS. This therapy comprised conventional compensatory and strengthening maneuvers (e.g., chin-tuck, Mendelsohn maneuver, and supraglottic swallow), individually tailored by the treating therapist. Because rehabilitation was delivered as part of routine clinical care in this retrospective cohort, its exact frequency and total duration were not systematically recorded and could not be retrieved; this continued therapy is acknowledged as an unavoidable co-intervention.

Patients were excluded if they had a history of invasive interventions for CPMD, including cricopharyngeal myotomy or balloon dilation, had undergone total laryngectomy, or had evidence of recent cancer recurrence. A minority of patients had also undergone surgery or flap reconstruction for a concurrent oral-cavity malignancy; because these procedures were anatomically remote from the cricopharyngeal region, they were not considered a direct cause of the cricopharyngeal dysphagia under study. A total of 18 patients meeting these criteria were consecutively enrolled prior to data analysis. The study was approved by the Institutional Review Board of our institution (IRB No. A-ER-114-193).

### 2.2. Botulinum Toxin Injection Procedure

All participants underwent endoscopy-guided injection of onabotulinumtoxinA (Botox^®^, Allergan Pharmaceuticals Ireland, Westport, Ireland), reconstituted with 0.9% sodium chloride to a concentration of 100 U/mL (100 units in 1 mL), targeting the cricopharyngeal muscle. The procedure followed a standardized protocol previously described, and no technical modifications were introduced for this analysis [17].

In brief, under topical anaesthesia a transnasal endoscope (ENF-VT2; Olympus, Tokyo, Japan) was introduced to the level of the larynx. A 16-Fr Foley catheter was then advanced transnasally through the upper esophageal sphincter into the cervical esophagus with the balloon deflated; the balloon was inflated with 10 mL of distilled water within the esophagus and gently withdrawn superiorly until it anchored against the inferior aspect of the cricopharyngeal–esophageal junction, thereby delineating the target muscle. Because the inflated balloon exceeded the caliber of the narrowed sphincter, it was not advanced or drawn through the cricopharyngeal segment but served solely as an inferior landmark; the technique was therefore not intended to dilate the upper esophageal sphincter. Through the working channel of a second transnasal endoscope (ENF TYPE T3; Olympus, Tokyo, Japan), an endoscopic puncture needle (Varix Puncture Needle; TOP Corporation, Tokyo, Japan) was used to inject botulinum toxin bilaterally into the proximal and distal portions of the cricopharyngeal muscle, giving a total of four injection sites, with each site receiving 5–10 units (0.05–0.1 mL at 100 U/mL). The total dose ranged from 20 to 40 units and was individualized according to three assessment-based criteria: the extent of cricopharyngeal narrowing observed during preoperative evaluation (larger affected areas warranting higher doses), the resting cricopharyngeal muscle tone (higher tension indicating a greater dose requirement), and the estimated cricopharyngeal muscle thickness (greater thickness suggesting a higher dose) [19,20,21].

### 2.3. Videofluoroscopic Swallowing Study

VFSS was performed before and after intervention to assess swallowing function [22,23]. Examinations were conducted using a fluoroscopy system (AXIOM Luminos dRF, Siemens, Munich, Germany) at a frame rate of 8–15 frames per second with patients in a seated position. For each swallow, patients received a single standardized bolus prepared by combining 3 mL of barium sulfate with 5 mL of thin liquid (International Dysphagia Diet Standardization Initiative level 0), administered by spoon. This same combined bolus was used for all trials and for every kinematic and temporal measurement (LE, LEV, and UES diameter); measurements were therefore not pooled across differing consistencies. The identical bolus preparation and administration protocol was applied at both the pre- and post-injection examinations. For the present analysis, VFSS recordings were reviewed frame by frame, and all measurements were performed using the built-in measurement tools of the picture archiving and communication system (INFINITT PACS, version 7.0.0.7; INFINITT Healthcare, Seoul, Republic of Korea) to enable precise temporal and kinematic assessment of laryngeal movement.

### 2.4. Outcome Measures

In the subjective assessment, the FOIS was used to evaluate the patient’s functional oral intake status. The FOIS is a 7-point ordinal scale that quantifies swallowing efficiency, with higher scores (1–7, 7 best) indicating better oral intake ability [24].

In the objective assessment, four parameters were used to assess swallowing physiology and airway protection: the PAS, LE, LEV, and UES diameter.

Airway invasion was evaluated using the 8-point PAS, which is based on the presence of penetration and aspiration and the cough response after food enters the airway. Higher scores indicate increased severity of laryngeal penetration or aspiration [25]. For each participant, the PAS score was defined as the worst score observed across all administered bolus trials [26]. This conservative, safety-oriented approach was adopted because airway invasion in this population does not necessarily occur on the first swallow; many patients have concurrent oral-phase impairment with slowed bolus transit and require several swallows to clear a single bolus, so aspiration may emerge only on later trials. Selecting the worst swallow therefore captures the maximum severity of airway invasion and reflects the most clinically meaningful risk.

LE was quantified as the vertical displacement of the larynx during swallowing, measured as the distance between the anterior–inferior corner of the tracheal air column at rest and at maximal superior excursion, as shown in Figure 1 [6]. To reduce the effects of anatomical variability and radiographic magnification, LE was normalized to the vertical distance between the anterior–inferior borders of the C2 and C4 vertebrae and expressed as C2–4% [27].

LEV was derived from this standardized displacement by incorporating the temporal dimension of movement. LEV was calculated by dividing normalized LE by the duration of the elevation phase, defined from the onset of upward laryngeal movement to the frame of maximal elevation, and expressed as C2–4%/s [6,27].

UES diameter was measured as the maximal anteroposterior distension of the sphincter during bolus passage, identified on the VFSS frame showing the widest opening in the sagittal plane [28]. This value was likewise normalized to the C2–4 vertebral distance.

Three otolaryngologists independently assessed all VFSS recordings, each blinded to the treatment condition (pre- versus post-botulinum toxin injection), to minimize assessment bias. All recordings were de-identified and randomized prior to review, and inter-rater discrepancies were resolved by consensus discussion.

### 2.5. Statistical Analysis

Baseline demographic and clinical characteristics were summarized using descriptive statistics. Continuous variables were reported as medians with interquartile ranges (IQRs), and categorical variables as frequencies and percentages.

Within-subject differences between pre- and post-intervention measurements were examined using the Wilcoxon signed-rank test. To address the primary objective, determining the association between LEV and swallowing function, Generalized Estimating Equations (GEEs) were utilized. This approach allowed for the evaluation of the predictive value of LEV while accounting for the within-subject correlation inherent in the repeated measures design. For the analysis of UES diameter, a linear GEE model with identity link function was constructed.

For airway safety, the PAS was dichotomized as “safe” (scores 1–4) versus “unsafe” (scores 5–8) and analyzed with a logistic GEE model (binomial distribution, logit link) [24,29,30,31], with LEV as the primary continuous predictor rescaled by a factor of 100 (LEV/100) to enhance interpretability. Dichotomizing the 7-level FOIS (“poor,” 1–3, versus “good,” 4–7) produced sparse outcome cells and unstable logistic-GEE estimates in this small sample; the FOIS was therefore assessed only with the Wilcoxon signed-rank test, and no dichotomized FOIS model is reported. To examine whether the association between LEV and UES diameter differed across pre- and post-injection states, an interaction term (LEV × Treatment) was incorporated into the multivariable linear GEE model for UES diameter. This interaction was specified a priori to test whether botulinum toxin injection was associated with a change in the biomechanical coupling between laryngeal kinematics and UES opening, reflecting a potential treatment-dependent modification of the traction mechanism governing sphincter distension. An analogous interaction term (LEV × Treatment) was also incorporated into the logistic GEE model for PAS to examine whether the association between LEV and airway safety differed according to treatment status. An exchangeable working correlation structure was assumed. All statistical tests were two-tailed, and a *p*-value of <0.05 was considered statistically significant. All analyses were performed using SAS 9.4 statistical software (SAS Institute Inc., Cary, NC, USA).

No a priori sample-size or power calculation was performed. Consistent with the exploratory, hypothesis-generating aim of this retrospective study, the sample size reflected the number of eligible patients available during the accrual window rather than a target derived from a formal power analysis. All findings are therefore interpreted as preliminary.

## 3. Results

### 3.1. Baseline Characteristics

This retrospective cohort study included 18 patients with NPC who developed CPMD after radiotherapy. The study population was predominantly male (77.8%) with a median age of 60 years. The majority of patients presented with advanced-stage disease (stage III or IV, 88.9%). All participants had undergone radiotherapy, and 83.3% had received concurrent chemotherapy. Regarding baseline functional status, 16.7% of the patients were dependent on tube feeding. The median interval between botulinum toxin injection and post-injection videofluoroscopic swallowing study was 56.5 days. The median interval between completion of radiotherapy and botulinum toxin injection was 10.6 years (IQR, 7.0–19.1; range, 2.6–30.2). Injection dosage was individualized according to anatomical and functional findings observed during preoperative assessment (median, 30 units; IQR, 22.5–37.5 units; range, 20–40 units). For detailed baseline characteristics, please refer to Table 1.

### 3.2. Pre–Post Comparisons of Swallowing Outcomes

Following the botulinum toxin injection, significant improvements were observed in key functional and kinematic parameters. The median FOIS score increased from 4 to 5 (*p* < 0.001), indicating an improvement in oral intake ability. The LEV demonstrated a significant increase from a median of 149.86 to 201.12 C2–4%/s (*p* = 0.027), and the UES diameter significantly widened from 21.10 to 23.30 C2–4% (*p* = 0.039). In contrast, changes in the PAS and LE were not statistically significant (Table 2).

### 3.3. Association Between LEV and UES Diameter

To investigate the dynamic relationship between laryngeal kinetics and sphincter opening, linear regression via GEE was performed (Table 3). In the univariate analysis, LEV was not significantly associated with UES diameter (*p* = 0.454), while the treatment effect (post- versus pre-injection) showed a significant positive association (*p* = 0.020). However, the interaction term (LEV × Treatment) revealed a significant interaction (*p* = 0.021), indicating that the association between LEV and UES diameter differed between pre- and post-treatment conditions.

To illustrate this interaction, the LEV–UES relationship was examined separately before and after injection (Figure 2). The Spearman correlation shifted from negligible and negative at baseline (ρ = −0.092) to moderate and positive after treatment (ρ = 0.340; *p* = 0.168), and the pre-treatment regression slope was negative (β = −0.034), consistent with the significant LEV × Treatment interaction identified in the GEE model (Table 3). Notably, the post-treatment GEE regression slope for LEV remained slightly negative rather than clearly positive; these descriptive correlations are presented to illustrate the modelled interaction rather than as an independent subgroup analysis.

### 3.4. Association Between LEV and Airway Safety (PAS)

Regarding airway safety as assessed by the PAS, the univariate analysis identified LEV as a significant predictor, with an odds ratio (OR) of 0.38 (95% confidence interval [CI], 0.17–0.86; *p* = 0.021). In the multivariable model adjusting for the treatment effect, LEV showed a trend toward an association with lower odds of unsafe airway invasion with an OR of 0.50 (95% CI, 0.24–1.04), although this did not reach statistical significance (*p* = 0.062). Upon introducing the interaction term (LEV × Treatment) into the model, the interaction effect was not statistically significant, and the main effects for both LEV and treatment also failed to reach significance (Table 4).

## 4. Discussion

This study represents the first preliminary investigation into the changes in LEV and their potential clinical implications in patients with NPC after RT complicated by CPMD, specifically assessing the effects of botulinum toxin injection. Our observational data indicate that (1) botulinum toxin injection was associated with statistically significant improvements in FOIS scores, LEV, and UES diameter; (2) LEV was associated with airway safety in univariate analysis, attenuated after adjustment, suggesting that velocity-based parameters may offer complementary, and potentially incremental, information beyond traditional displacement measures in characterizing airway protection; and (3) observed data patterns are consistent with the possibility that botulinum toxin treatment may be associated with a modulation in the biomechanical coupling between laryngeal motion and UES opening, though this interpretation requires further validation.

### 4.1. LEV and Swallowing Safety

In univariate GEE analysis, LEV was associated with airway safety; for every 100-unit (C2–4%/s) increase in LEV, the odds of an “Unsafe” PAS outcome decreased by 62% (univariate model), although this association was attenuated and no longer significant after adjustment for treatment status. This observation aligns with findings from one previous study in acute ischemic stroke patients, where reduced laryngeal elevation velocity was identified as an independent predictor of aspiration risk [6]. Notably, in our cohort, the maximal distance of LE did not exhibit significant changes pre- and post-treatment (*p* = 0.580), suggesting that displacement amplitude alone may be insufficient to capture the biomechanical determinants of airway protection in this population [11]. LEV may therefore reflect the neuromuscular system’s capacity to generate the rapid force required for effective airway protection.

Importantly, although LEV was associated with airway safety in pooled analyses, PAS scores did not improve significantly after the perturbation (*p* = 0.695). This association should therefore be interpreted as a cross-sectional relationship between kinematics and airway status across observations, rather than as evidence that botulinum toxin injection improved airway protection. Accordingly, LEV is best regarded here as a candidate marker of swallowing safety rather than as a validated therapeutic target.

### 4.2. Potential Impact of Botulinum Toxin on the Dynamic LEV–UES Relationship

The significant LEV × Treatment interaction on UES diameter (Table 3) suggests that botulinum toxin injection may provide a context in which altered biomechanical coupling between laryngeal motion and UES opening becomes observable [7]. The descriptive pre- and post-treatment correlations presented in the Results are directionally consistent with this interpretation; however, the post-treatment association did not reach statistical significance and the GEE regression slope for LEV remained slightly negative, so the interaction is most conservatively interpreted as an attenuation of the pre-treatment negative coupling rather than a reversal to effective positive coupling. This pattern should therefore be regarded as hypothesis-generating and requires confirmation in larger studies.

In normal swallowing, UES opening relies partly on traction forces generated by hyolaryngeal elevation [8,32]. In post-radiotherapy nasopharyngeal carcinoma patients, fibrosis or hypertonicity of the cricopharyngeal muscle may increase sphincter resistance, resulting in mechanical decoupling between laryngeal elevation and UES opening [7,15]. This pathological state is consistent with the negative pre-treatment association between laryngeal elevation and UES distension observed in our GEE model, in which greater laryngeal elevation was not effectively translated into sphincter opening; however, this mechanistic interpretation remains speculative given the observational design. A plausible interpretation is that reduced cricopharyngeal muscle tone following chemical denervation may lessen this resistance, potentially allowing traction forces to be more effectively translated into UES opening. This proposed mechanism is speculative and cannot be confirmed by the present observational, uncontrolled data.

### 4.3. Biomechanical Considerations for Changes in LEV

The observed increase in LEV following intervention, despite no significant change in laryngeal elevation displacement, may reflect an adaptive response of the hyolaryngeal system to altered biomechanical loading conditions rather than a direct treatment-specific effect. In patients with post-radiotherapy cricopharyngeal muscle dysfunction, increased sphincter resistance may constrain laryngeal motion, limiting elevation velocity even when final displacement is preserved. When this resistance is reduced, the same neuromuscular activation may result in faster elevation without altering excursion magnitude. From this perspective, LEV represents a load-sensitive kinematic parameter that captures functional efficiency of laryngeal elevation under varying mechanical constraints, complementing traditional displacement-based measures [33]. Importantly, changes in LEV should not be interpreted as direct evidence of neuromuscular recovery, but rather as emergent kinematic behavior under altered mechanical loading conditions.

### 4.4. Interpretation of the FOIS Improvement

FOIS improved significantly after the perturbation (median 4 to 5; *p* < 0.001). This functional gain is likely multifactorial rather than attributable to any single kinematic parameter. FOIS reflects global daily dietary function, which is shaped not only by physiological factors such as LEV and airway status but also by oral-phase control, sensory feedback, and patient confidence. The broader set of changes accompanying the perturbation (e.g., reduced residue, decreased effort) may therefore account for the change in diet levels more than LEV alone; whether these reflect a specific treatment effect cannot be determined from this uncontrolled design.

### 4.5. Limitations

This study has several limitations. First, the small, single-center sample (N = 18) was determined by patient availability rather than an a priori power calculation, so all analyses are exploratory; limited power increases the risk of Type II errors, and multiple comparisons were performed without formal correction. GEE’s robust variance estimator is downward-biased with few clusters, so with N = 18 the standard errors, especially for the interaction models, may be underestimated and those estimates should be viewed as unstable and hypothesis-generating. Because the botulinum toxin dose was individualized to disease severity, it is collinear with baseline severity and was not entered as a covariate to avoid overadjustment given the limited events-per-variable ratio.

Second, the single-arm design without a concurrent control group means that regression to the mean and non-specific adaptive effects cannot be fully excluded, although the chronic, rehabilitation-refractory nature of the cohort makes spontaneous recovery an unlikely sole explanation. Individual radiotherapy dose, fractionation, and technique were unavailable, and the cohort was oncologically heterogeneous; nonetheless, all patients were diagnosed by an otolaryngologist and presented with comparable dysphagia severity, providing a degree of clinical homogeneity. Both the radiotherapy-to-injection interval and the injection-to-VFSS interval were variable, introducing heterogeneity in chronicity and treatment timing, and swallowing rehabilitation continued throughout as an unavoidable co-intervention. In addition, although the localization balloon was anchored inferior to the sphincter and was not passed through it, a minor mechanical effect from transient traction on the radiation-fibrosed segment cannot be entirely excluded.

Third, videofluoroscopy was acquired at 8–15 frames per second owing to institutional equipment configuration; although this affects a velocity-based measure more than a displacement-based one, each patient’s pre- and post-injection studies used the same frame rate, so systematic timing bias should largely offset within the paired comparisons. Finally, inter-rater reliability was not formally quantified—consensus review resolved discrepancies but does not itself measure reliability, and per-rater scores were not retained, precluding post hoc intraclass correlation or weighted kappa.

## 5. Conclusions

In this exploratory study of NPC patients who developed CPMD after RT, LEV showed an association with swallowing safety and a shift in its relationship with UES opening across pre- and post-injection states. Unlike displacement-based measures, LEV appears to capture load-sensitive aspects of hyolaryngeal kinematics relevant to airway protection. Although the botulinum toxin perturbation was accompanied by functional and kinematic improvements, the study design does not permit conclusions about treatment efficacy; the present findings therefore position LEV primarily as a candidate marker of swallowing efficiency under altered mechanical conditions, rather than as an indicator of neuromuscular recovery or a validated clinical biomarker. Confirmation in larger, prospective studies is required before any clinical application.

## Figures and Tables

**Figure 1:**
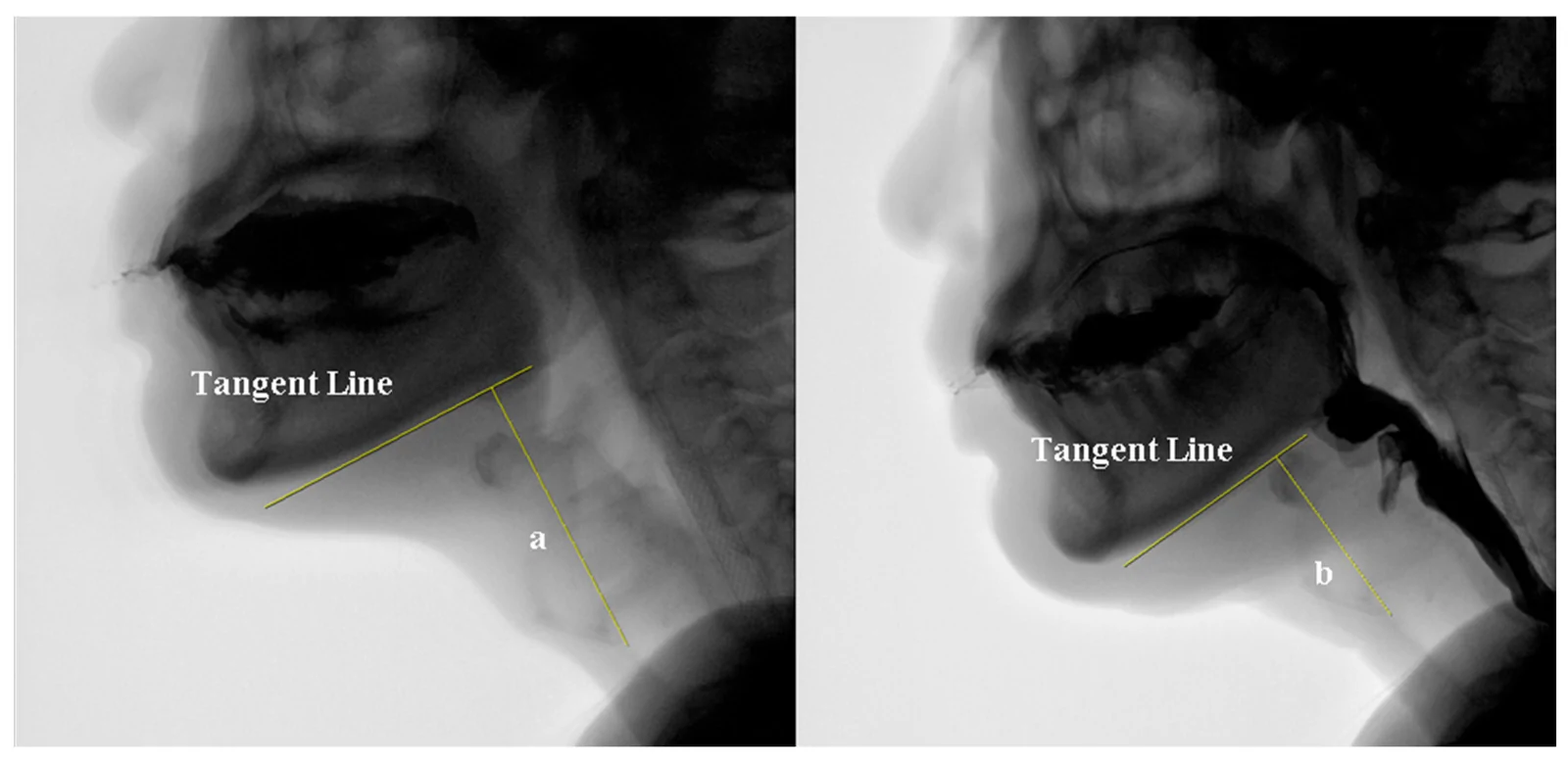
Measurement of Laryngeal Elevation and Laryngeal Elevation Velocity. The tangent line indicates the tangent to the inferior border of the mandible. Distance a represents the perpendicular distance between the mandible and the anterior corner of the laryngeal air column at rest. Distance b represents the corresponding distance measured at the point of maximal superior laryngeal elevation during swallowing. Laryngeal elevation was calculated as the difference between distances b and a.

**Figure 2:**
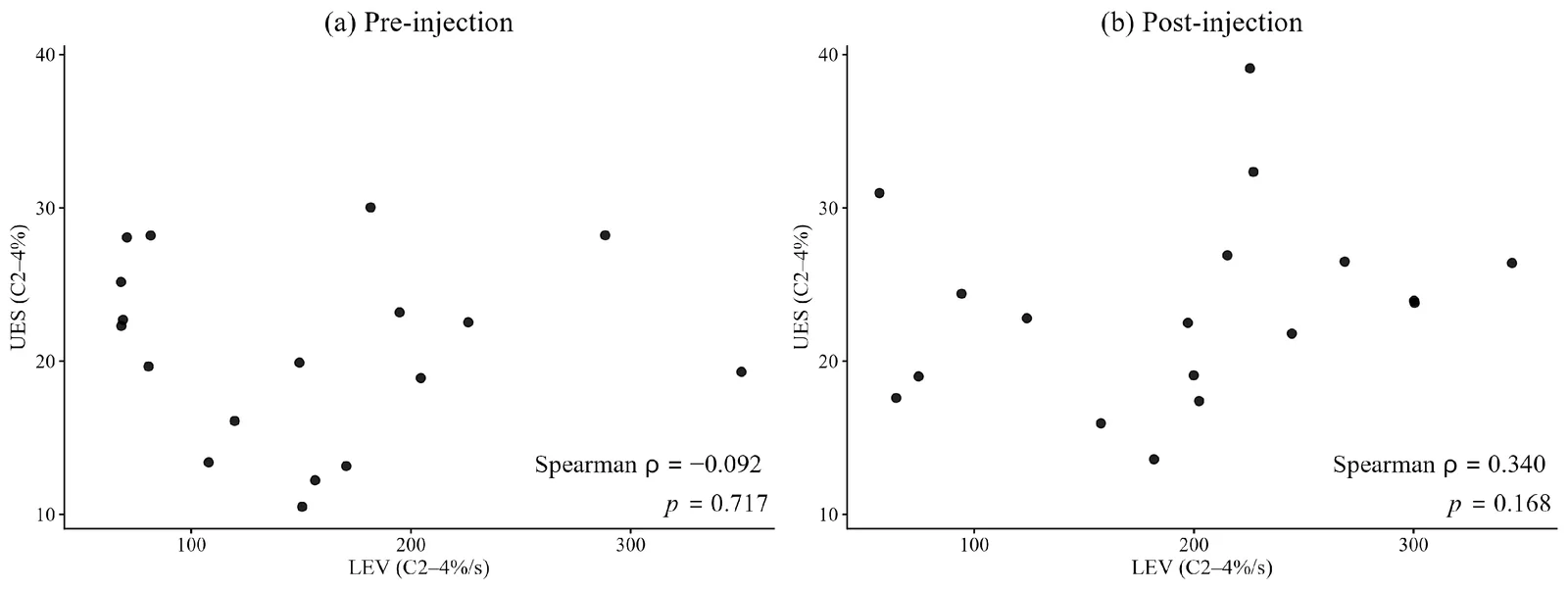
Relationship between LEV and UES diameter before and after botulinum toxin injection. (**a**) Pre-injection; (**b**) post-injection. Each dot represents one patient; Spearman’s rank correlation coefficient (ρ) and *p*-values are shown in each panel. Abbreviations: LEV = laryngeal elevation velocity; UES = upper esophageal sphincter; C2–4% = percentage of the C2–C4 vertebral distance.

**Table 1:** Characteristics of included patients.

	N	
N	18	
Male	14	77.8%
Age (years), median (IQR)	60.0	(51.0–66.0)
NPC stage		
1	0	0%
2	2	11.1%
3	10	55.6%
4	6	33.3%
Treatment		
surgery	2	11.1%
flap implantation	1	5.6%
chemotherapy	15	83.3%
radiotherapy	18	100%
NG tube/ND tube/PEG placement	3	16.7%
Duration (days) between botulinum toxin injection and post-injection VFSS examination, median (IQR)	56.5	(22, 163)
Duration (years) between radiotherapy completion and botulinum toxin injection, median (IQR)	10.6	(7.0, 19.1)

Abbreviations: NPC = Nasopharyngeal carcinoma; NG = nasogastric; ND = Nasoduodenal; PEG = percutaneous endoscopic gastrostomy.

**Table 2:** Differences between pre-intervention and post-intervention.

	Pre *		Post		
	Median	IQR	Median	IQR	*p*
FOIS	4	(4, 5)	5	(4, 6)	<0.001
PAS	5	(1, 6)	4	(1, 7)	0.695
LE	36.83	(26.99, 43.81)	41.26	(28, 53.96)	0.580
LEV	149.86	(80.54, 194.83)	201.12	(123.95, 244.59)	0.027
UES	21.10	(16.80, 24.67)	23.30	(19.02, 26.48)	0.039

*: Relative to botulinum toxin injection. Abbreviations: FOIS = functional oral intake scale; PAS = penetration–aspiration scale; LE = laryngeal elevation; LEV = laryngeal elevation velocity; UES = upper esophageal sphincter.

**Table 3:** Linear regression via generalized estimating equations (GEEs) for UES.

	Outcome: UES								
	Univariate			Multivariable			Multivariable		
	β	SE	*p*	β	SE	*p*	β	SE	*p*
LEV/100	0.813	1.030	0.454	−0.847	1.132	0.440	−3.418	1.596	0.019
Treatment (post vs. pre)	2.810	1.208	0.020	3.274	1.108	0.004	−1.836	2.547	0.475
LEV/100 × treatment							3.117	1.463	0.021

Abbreviations: LEV = laryngeal elevation velocity; UES = upper esophageal sphincter; SE = standard error.

**Table 4:** Logistic regression via generalized estimating equations (GEEs) for PAS unsafe (PAS:5–8).

	Outcome: PAS								
	Univariate			Multivariable			Multivariable		
	OR	95% CI	*p*	OR	95% CI	*p*	OR	95% CI	*p*
LEV/100	0.38	(0.17, 0.86)	0.021	0.50	(0.24, 1.04)	0.062	0.62	(0.31, 1.24)	0.175
Treatment (post vs. pre)	0.51	(0.25, 1.04)	0.063	0.63	(0.29, 1.33)	0.224	1.18	(0.51, 2.73)	0.705
LEV/100 × treatment							0.68	(0.42, 1.10)	0.118

Abbreviations: LEV = laryngeal elevation velocity; PAS = penetration–aspiration scale; OR = odds ratio; CI = confidence interval.

## Data Availability

Due to data privacy restrictions, the data supporting the findings of this study are not publicly available.

## Abbreviations

The following abbreviations are used in this manuscript:

LEV	Laryngeal Elevation Velocity
UES	Upper Esophageal Sphincter
NPC	Nasopharyngeal Carcinoma
CPMD	Cricopharyngeal Muscle Dysfunction
VFSS	Videofluoroscopic Swallowing Study
PAS	Penetration–Aspiration Scale
FOIS	Functional Oral Intake Scale
IQRs	Interquartile Ranges
GEEs	Generalized Estimating Equations
CI	Confidence Interval
LE	Laryngeal Elevation
LVC	Laryngeal Vestibule Closure
RT	Radiotherapy
IRB	Institutional Review Board
OR	Odds Ratio
SE	Standard Error
NG	Nasogastric (tube)
ND	Nasoduodenal (tube)
PEG	Percutaneous Endoscopic Gastrostomy
